# Extra Hepatic Biliary Atresia Associated with Choledochal Cyst: A Diagnostic Dilemma in Neonatal Obstructive Jaundice

**Published:** 2013-01-01

**Authors:** Shalini Sinha, Yogesh Kumar Sarin

**Affiliations:** Department of Pediatric Surgery, Maulana Azad Medical College, New Delhi-110002

**Keywords:** Extra hepatic biliary atresia, Choledochal cyst, Neonatal obstructive jaundice

## Abstract

The presentation of extra hepatic biliary-atresia (EHBA) as well as choledochal cyst (CDC) in the neonate may be similar. Since the surgical management and prognosis are entirely different, it is important to differentiate between the two entities. We present a case with co-existing EHBA and CDC which led to a diagnostic dilemma.

## INTRODUCTION

Surgical causes of neonatal obstructive jaundice include extra hepatic biliary atresia (EHBA) and rarely infantile form of choledochal cyst (CDC) [1]. Since the management approaches and prognoses are dramatically different, it is important to differentiate between the two entities. We present a case of type 3 EHBA (The Japanese Association of Pediatric Surgeons classification) with co-existing Type I CDC (Todani) which led to a diagnostic dilemma.

## CASE REPORT

A 45-day-old boy presented with progressively increasing neonatal obstructive jaundice and acholic stools. He was born by normal vaginal delivery at term with a birth weight of 2500 g and normal APGARs. The pregnancy was supervised and uneventful with two normal prenatal ultrasounds. There were no maternal risk factors identified. The neonate was well till day 3 of life when the parents noticed yellowish discoloration of the sclera followed by pale colored stools. He was evaluated and treated by several pediatricians in his hometown, without any avail, before being referred to us. There were no symptoms of bleeding diatheses. His general condition was well preserved but he was deeply icteric. He had a firm liver palpable 3 cm below the costal margin. Liver function tests (LFT) showed mildly deranged coagulation profile with a total bilirubin of 14.5 and direct bilirubin of 7.8mg/dl, elevated liver enzymes and normal proteins. The fasting ultrasonography revealed an enlarged liver with a normal gall bladder (GB) and common bile duct (CBD). There was no evidence of any subhepatic cyst but postprandial contraction of the GB could not be demonstrated. His TORCH titres were positive for Cytomegalovirus (CMV) IgM antibodies; however urine sample was negative for PCR detection of CMV, thus ruling out congenital CMV infection. A HIDA scan after 5 days of oral phenobarbitone revealed no excretion of radioisotope into the gut after 24hrs. Liver biopsy and MRCP were not done. Since the preoperative evaluation was highly suggestive of type 3 EHBA, he was taken up for laparotomy on day 54 of life without any further delay. At laparotomy, the liver was found to be cirrhotic. A normal looking GB, 2.7 cms long, was found to be communicating with a 4 x 4 cms cyst replacing the CBD (Fig. 1). 

**Figure:**
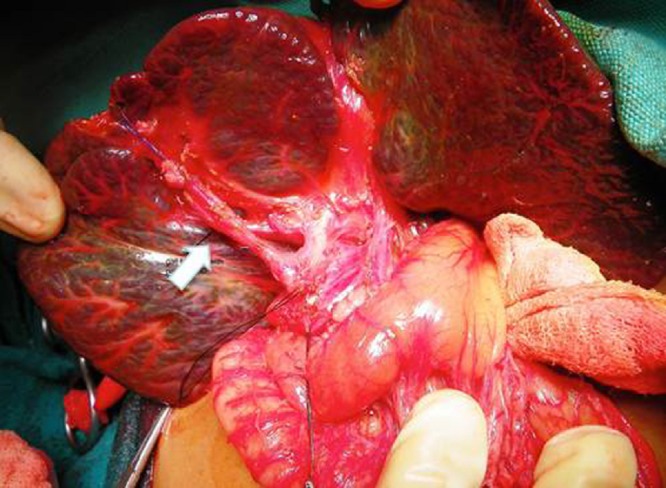
Figure 1: Intraoperative picture showing a cirrhotic liver with a long gall bladder (block arrow) and patent CBD.

Needle aspiration did not reveal any bile. The above findings were confirmed by intra-operative cholangiography which demonstrated the cystic duct opening into a cystic structure similar to a CDC. The intra-hepatic ducts were not visualized and there was no dye in the duodenum (Fig. 2).

**Figure:**
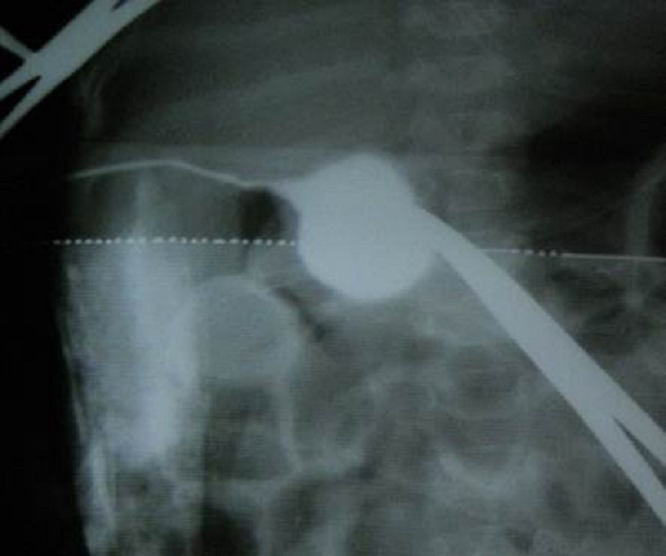
Figure 2: Peroperative cholangiogram showing gall bladder draining into CDC with lack of opacification of proximal hepatic ducts and no dye seen entering the duodenum.

The cyst was unlikely to be a bile lake as it was in continuity with the GB and extra hepatic in location. On further exploration, the common hepatic duct (CHD) was found obliterated just at its origin and no bile flow was seen. The CDC was excised using Lilly's technique and Kasai's portoenterostomy (KPE) was done using a 20 cms long Roux loop of jejunum. Histopathology confirmed both EHBA and CDC with evidence of liver cirrhosis and periportal fibrosis. The atretic plate showed multiple bile ducts and the CDC had chronic inflammation of the cyst wall. The child recovered and was passing cholic stools from the 4th postoperative day with significant improvement in LFT (Total bilirubin of 7.5 and direct 4.5 mg/dl, alkaline phosphatase 190 IU/L). He was tolerating oral feeds well and was discharged on the 13th postoperative day on oral chemoprophylaxis, prednisolone, ursodeoxycholic acid and fat soluble vitamin supplements. He was followed up at 2 months with one episode of cholangitis which was treated conservatively with 4 weeks of 2nd line intravenous antibiotics. Within a month of discharge from hospital, he developed another episode of cholangitis and unfortunately died at home, which was 200 Kms away from our hospital, before treatment could be reinstated.

## DISCUSSION

A thorough literature search revealed less than 100 cases of EHBA associated with CDC reported till date [2-8] of which majority were Type 1-EHBA (76%) and only 21% were Type 3-EHBA (our case) [2]. CDC associated with Type 1-EHBA has been described only in neonates who are also found to have distal atresia of the CBD during intra-operative cholangiogram [3]. EHBA with CDC should be differentiated from Type 1-cystic EHBA which also has a prenatal origin but the fibro-inflammatory cyst wall does not have biliary epithelial lining nor does it have continuity with the biliary tree [9]. 

As regards to the etiopathogenesis of CDC, the well accepted Babbitt’s hypothesis of anomalous pancreaticobiliary union resulting in acquired CDC holds true only for children and young adolescents and cannot explain the presentation of neonatal/ infantile CDC [10]. In 1974, Landing hypothesized that neonatal hepatitis, biliary atresia, and choledochal cyst represented a spectrum of manifestations of what he called “infantile obstructive cholangiopathy”. He proposed that the panductular sclerosis of EHBA and the extrahepatic distal obstruction responsible for the formation of the CDC were due to a perinatal infectious cholangitis (most likely of viral origin), which progressed to a fibrotic scarring of the biliary tract [11]. Only Landing's hypothesis can explain the possible etiopathogenesis of coexisting EHBA and CDC. 

A noticeable finding was the early onset of jaundice in this baby (day 3 of life). This is usually seen in Biliary Atresia Splenic Malformation (BASM) (polysplenia, situs inversus and portal vein anomalies) syndrome which has an incidence of 10-15% [2, 5, 12]. EHBA associated with CDC has also been shown to present at birth and even diagnosed antenatally, though the prognosis is better than that of BASM [5]. 

As we are all aware, no single preoperative investigation can diagnose EHBA with cent percent accuracy. Surgical exploration and intra-operative cholangiography form the mainstay of confirmatory diagnosis of EHBA. A good ultrasound by an experienced and focussed radiologist can provide valuable information in the workup of neonatal obstructive jaundice. Humphrey et al have demonstrated the following highly sensitive and specific ultrasound features for preoperative diagnosis of EHBA - triangular cord sign (sensitivity 73% and specificity 100%), abnormal gallbladder wall (sensitivity 91% and specificity 95%) and shape (sensitivity 70% and specificity 100%), and an absent common bile duct (sensitivity 93% and specificity 92%). In addition, they postulated for the first time, that jaundiced infants with EHBA had a significantly larger hepatic artery diameter when compared to those without EHBA [13]. These findings have been simulated by other authors [14]. 

The most pertinent sonographic finding in a case of CDC is a well defined cyst below the portahepatis with or without intrahepatic biliary radicle dilatation. In present case, the 4 x 4 cms subhepatic cyst was missed on ultrasonography though the patent CBD was identified, classifying the EHBA as Type 3. The combination of a detailed ultrasonogram and hepatic scintigraphy should raise the preoperative suspicion of EHBA associated with CDC as the subhepatic cyst seen on sonography will not accumulate the hepatic radioisotope on scintigraphy nor will there be any intestinal activity seen. Moreover, an experienced and aware sonologist can differentiate between EHBA associated with CDC, and infantile CDC, by the larger diameter of the cysts, presence of intra-hepatic biliary dilatation and normal gall bladders in the latter group [1]. 

Infantile CDC should also be distinguished from bile lakes which are usually located in the central part of the liver and are seen in long standing cirrhohis/ cholestasis with or without EHBA. They are pseudocysts, lacking an epithelial lining and do not communicate with the biliary tree. They can also be seen after KPE for EHBA [15]. 

MRCP is an additional tool in our armamentarium for the diagnostic workup of infantile CDC. Liver biopsy can help in differentiating EHBA from neonatal hepatitis but it is an invasive procedure and may be complicated with bleeding. Since the patients in our setup often present late, we do not routinely perform preoperative liver biopsy (which would take another week to get reported at our institute). With a negative workup for medical causes of neonatal jaundice, and failure of excretion of hepatobiliary radionucleotide into the intestine on scintigraphy even at 24 hours; our protocol is to proceed with laparotomy and intraoperative cholangiogram which was done in this case. 

The importance of per operative cholangiogram, which is still the gold standard for diagnosis of EHBA, cannot be overemphasized. We were able to identify this rare coexistence during surgery only because of the findings on cholangiography. Vice versa, absence of bile flow during surgery for CDC should also raise the suspicion of coexisting proximal EHBA which can easily be missed on naked eye examination [8]. Another important intra-operative finding is an abnormal gall bladder (atretic or hypoplastic) during surgery for CDC. Often these abnormalities may not be appreciated on ultrasonography. If the gall bladder is abnormal, intra-operative cholangiography is a must to rule out proximal or distal EHBA [3]. Even though, in our case, the gall bladder looked normal and was 2.7 cms long. 

The outcome is usually similar to that of EHBA if diagnosed and operated on time. Delay in diagnosis increases the chances of failure. It is unfortunate that though the KPE was functioning, we lost this child to a completely treatable etiology like cholangitis.

EHBA associated with CDC is a rare cause of neonatal obstructive jaundice. Combined interpretation of a focussed ultrasound and hepatic scintigraphy can diagnose this condition preoperatively. We recommend intra-operative cholangiography for all cases of either EHBA or infantile CDC. The presence of an abnormal gall bladder and absence of bile flow during surgery for CDC should prompt the surgeon to further explore the possibility of proximal biliary atresia.

## Footnotes

**Source of Support:** Nil

**Conflict of Interest:** One of the authors’ belongs to editorial board; however, the manuscript was independently handled by other editors. The authors are not involved in any decision making regarding this manuscript.
